# The Moderating Role of Caregivers’ Personality Traits in the Relationship Between Dementia Severity and Caregiver Burden

**DOI:** 10.1002/alz70858_104183

**Published:** 2025-12-25

**Authors:** So Yeon Jeon, JiHye Heo, Hee Won Yang, Jeong Lan Kim, Dong Hun Lee

**Affiliations:** ^1^ School of Medicine, Chungnam National University, Daejeon, Korea, Republic of (South); ^2^ Chungnam National University Hospital, Daejeon, Daejeon, Korea, Republic of (South); ^3^ Department of Biomedical Engineering Chungnam National University, Daejeon, Korea, Republic of (South); ^4^ Chungnam National University Hospital, Daejeon, Korea, Republic of (South); ^5^ Department of Radiology, College of Medicine, Seoul National University, Seoul, Korea, Republic of (South)

## Abstract

**Background:**

Caregiving for individuals with dementia often results in significant psychological and emotional burden, which varies widely among caregivers. Personality traits are critical factors that may influence how caregivers perceive and cope with caregiving challenges, potentially moderating the impact of dementia severity and behavioral symptoms on caregiver burden. This study aims to examine these moderating effects, providing insights to inform targeted interventions for caregivers.

**Method:**

We analyzed 215 caregiver‐care recipient dyads from Chungnam National University Hospital. Dementia severity (CDR), behavioral symptoms (NPI), caregiver burden (ZBI), and caregiver personality (BFI‐5) were assessed. Moderation analyses included covariates (age, sex, cohabitation)

**Result:**

Care recipients had a mean age of 77.04 ± 7.06 years (42.33% female), while caregivers had a mean age of 65.23 ± 17.34 years (61.9% female). Among caregivers, 91 were spouses, 104 were children, and 20 were other relatives. Global CDR was significantly associated with ZBI_total (β = 14.678, *p* < .001). Extraversion (β = ‐6.837, *p* = .047) and neuroticism (β = 7.594, *p* = .023) showed significant moderating effects. Simple slope analysis revealed that the effect of global CDR on ZBI_total was stronger at low extraversion (‐1SD, β = 17.8, *p* < .001) compared to high extraversion (+1SD, β = 11.7, *p* < .001), and stronger at high neuroticism (+1SD, β = 17.36, *p* < .001) compared to low neuroticism (‐1SD, β = 8.87, *p* = .001). Other personality traits, including conscientiousness, openness, and agreeableness, did not show significant moderating effects. NPI severity × frequency was significantly associated with NPI distress (β = 0.982, *p* < .001); however, no personality traits moderated this relationship.

**Conclusion:**

These findings highlight the moderating effects of extraversion and neuroticism on the relationship between dementia severity and caregiver burden, with higher extraversion buffering and higher neuroticism amplifying the burden. Additionally, BPSD severity strongly predicted caregiver distress, independent of personality traits. These results underscore the need to incorporate personality assessments into caregiver support programs to address individual differences and improve outcomes in dementia care.

